# Sensitive Detection of Epidermal Growth Factor in Lung Cancer Patients by Electrochemical Biosensors

**DOI:** 10.1021/acs.analchem.6c01773

**Published:** 2026-04-24

**Authors:** Tatiana Lima Valério, Bruna M. Hryniewicz, Fernanda Luisa Basei, Mel De Souza Wendhausen Araújo, Hussamaldeen Jaradat, Olfa Kanoun, Flavia Raquel Gonçalves Carneiro, Tatiane Caldas Montella, Carlos Gil Moreira Ferreira, Nilson Ivo Tonin Zanchin, Marcio Vidotti

**Affiliations:** † Grupo de Pesquisa em Macromoléculas e Interfaces, 28122 Federal University of Paraná (UFPR), 81531-980, Curitiba, PR, Brazil; ‡ Departamento de Físico-Química, Instituto de Química, Universidade Federal da Bahia, Salvador, Bahia 40170-115, Brazil; § Instituto Carlos Chagas − Fiocruz, 81310-020, Curitiba, PR, Brazil; ∥ Professorship of Measurement and Sensor Technology, Faculty of Electrical Engineering and Information Technology, Chemnitz University of Technology, 09126 Chemnitz, Germany; ⊥ Center for Technology Development in Health (CDTS), 37903 FIOCRUZ, Rio de Janeiro 21040-900, RJ, Brazil; # Interdisciplinary Laboratory of Medical Research, Oswaldo Cruz Institute (IOC), FIOCRUZ, Rio de Janeiro 21040-900, RJ, Brazil; ∇ Program of Immunology and Tumor Biology, Brazilian National Cancer Institute (INCA), Rio de Janeiro 20231-050, RJ, Brazil; ○ Oncoclínicas, Rio de Janeiro 22250-905, Brazil

## Abstract

Lung cancer is the most common cancer worldwide, responsible for more deaths per year than any other cancer, with non-small cell lung cancer (NSCLC) being the most prevalent form. Elevated epidermal growth factor (EGF) levels have been associated with NSCLC and decreased survival, highlighting its potential as a prognostic and predictive biomarker. This study proposes developing and validating a novel impedimetric electrochemical biosensor for the sensitive and accurate detection of EGF in serum samples from patients with lung adenocarcinoma. The biosensor was constructed by electropolymerization of polypyrrole nanotubes (PPy-NTs) on screen-printed carbon electrodes, followed by the deposition of carboxylated multiwalled carbon nanotubes (MWCNT-COOH) used for the immobilization of biorecognition elements. Anti-EGF antibodies were covalently immobilized on the MWCNT-COOH-modified surface, and bovine serum albumin (BSA) was used to block nonspecific interactions. Electrochemical impedance spectroscopy (EIS) was employed to characterize each fabrication step and to detect EGF. The biosensor demonstrated a wide linear detection range from 500 fg mL^–1^ to 200 pg mL^–1^ with high linearity (*R* ^2^ = 0.9924) and a remarkably low limit of detection (LOD) of 392 fg mL^–1^. The developed biosensor demonstrated excellent agreement with ELISA results, with a strong Pearson correlation (*r* = 0.865, *p* < 0.0001), and Bland-Altman analysis with 97.4% of the differences within the limits of agreement. The biosensor successfully monitored changes in EGF levels in patients undergoing chemotherapy, showing a percentage change similar to that observed with ELISA. These results indicate that the developed impedimetric biosensor is a promising, sensitive, and reliable alternative for monitoring EGF levels in lung cancer patients, potentially aiding in prognosis and treatment evaluation.

## Graphical abstract

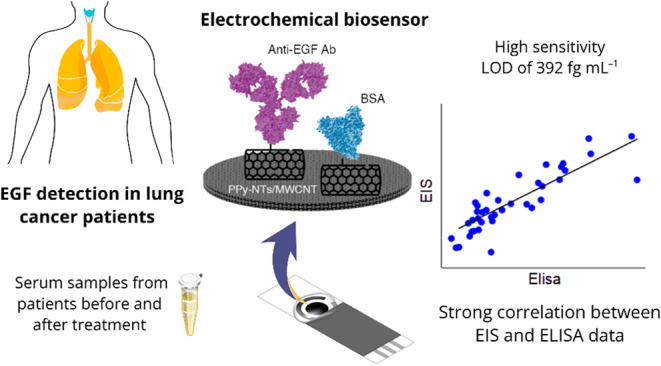

## Introduction

Lung cancer is the leading cause of cancer-related deaths worldwide. Approximately 2.5 million new cases and 1.8 million lung cancer-related deaths are estimated annually.^1^ The overall 5-year survival rate is only 15.8% and the majority of individuals, approximately 78%, are diagnosed with metastatic or advanced disease.^1,2^ Nonsmall cell lung cancer (NSCLC) is the most prevalent form of lung cancer, representing approximately 85% of all cases.^3^

In cancer, one of the mechanisms that enables the proliferation of malignant cells is the alteration of cellular signaling mechanisms, caused by the overexpression of growth factors or by the overexpression and autoactivation of cellular receptors. Cells acquire the ability to proliferate when different ligands, such as epidermal growth factor (EGF), bind to the extracellular domains of their receptors, initiating an intracellular signaling cascade.^4–6^ Dysregulated activation of the epidermal growth factor receptor (EGFR) pathway occurs due to different factors, including overexpression of the receptor itself, overproduction of ligands, and the appearance of oncogenic mutations that induce constitutive activation of the receptor. Studies suggest that receptor overexpression often occurs in the presence of overproduction of ligands.^7^

Although there is not a clear consensus in the scientific community about the correlation of EGF ligand levels in NSCLC patients compared to healthy individuals,^8,9^ more recent research has demonstrated that elevated levels of EGF are associated with NSCLC^10–12^ and that patients with elevated serum EGF levels have a shorter survival rate compared with those with lower levels, even under similar conditions. EGF overexpression is often associated with aggressive tumor characteristics, including increased proliferation, invasiveness, and metastasis. Therefore, the presence of high serum EGF levels may be correlated with advanced stage disease, reflecting the negative impact of tumor burden on patient prognosis.^5,13^

In this sense, different alternatives have been studied to block receptor activation by targeting EGF instead of EGFR. One example is the study of the CIMAvax-EGF vaccine, considered the first vaccine developed against EGF.^5,13,14^ Phase IV clinical trials were completed^3^ and the vaccine is already available in various countries. This vaccine induces an immune response to produce effector antibodies against EGF, thereby reducing its availability and disrupting the signaling pathways that promote cancer cell survival and proliferation, especially in patients already identified with high levels of EGF. This suggests that evaluating EGF levels may be important for prognosis and identifying patients who may benefit most from vaccination with CIMAvax-EGF.^5,13^ Moreover, EGF has been implicated in various other conditions, including various types of cancer^15–19^ Parkinson’s disease,^20^ chronic kidney disease,^21^ lung disorders^22^ and hepatitis B virus (HBV) infection,^23^ highlighting the importance of accurate EGF detection.

Antibodies and antibody fragments targeting EGF have been studied for their potential use in clinical assays.^7^ Monitoring EGF levels using anti-EGF antibodies is usually done through ELISA-type tests; however, new strategies have been developed for faster and more sensitive detection, such as the construction of electrochemical biosensors, which offer high sensitivity, selectivity, and ease of application.^24–28^ Despite these advantages, to the best of our knowledge, this is the first study to explore the detection of EGF levels in lung adenocarcinoma patients using a biosensor-based platform, highlighting the potential of this analytical tool to support future investigations into the role of EGF in disease progression and therapeutic monitoring. It is important to note that this work specifically investigates EGF in the context of lung cancer. Therefore, the interpretation of EGF levels should be considered within the appropriate clinical context and in combination with other diagnostic approaches. In this sense, the proposed biosensor is intended to contribute as a complementary analytical tool for screening and monitoring purposes rather than as a standalone diagnostic method.

Different types of electrochemical biosensors have been studied, with those based on the electrochemical impedance spectroscopy (EIS) technique standing out, as they present high sensitivities and low detection limits.^29–31^ In this method, the detection of the biomolecule is associated with differences in the charge transfer resistance at the electrode/electrolyte interface. After the antigen–antibody interaction, the charge transfer resistance increases due to the immobilization of insulating molecules, with the extent of the increase proportional to the amount of immobilized biomolecules.^29^

The use of conductive polymers in biosensors is highly advantageous, presenting high conductivity, excellent cost-benefit, biocompatibility, high sensitivity, and stability. In addition, conductive polymers present oxidation/reduction reactions, which lead to the appearance of charge transfer resistance, crucial for the detection of biomolecules, making the use of additional redox probes unnecessary.^29,32^

Multiwalled carbon nanotubes functionalized with carboxyl groups (MWCNT-COOH) have been widely used in the immobilization of biorecognition elements. Their carboxyl groups (−COOH) enable the covalent anchoring of antibodies via activation with EDC/NHS, providing greater stability and specificity in biomolecular detection. This approach allows the construction of electrochemical biosensors directly and efficiently, without the need for linkers or additional additives.^1,30^

To date, no studies have been found in the literature exploring the electrochemical detection of EGF levels in the blood of lung adenocarcinoma patients using anti-EGF antibodies. Thus, this work proposes an approach for monitoring lung cancer biomarkers in serum before and after treatment, contributing significantly to the evaluation of the efficacy of new specific antineoplastic compounds.

## Materials and Methods

### Biosensor Construction

The electrochemical procedures were performed using the Metrohm Autolab PGSTAT204 equipment. Initially, screen-printed carbon electrodes (SPEs) containing a carbon working electrode, a carbon counter electrode and a silver pseudoreference electrode, Metrohm Brasil, were cleaned by polishing with aluminum oxide dispersion and then electrochemically activated according to the method described by Rahmati et al.^33^ The monomer electropolymerization was performed according to Hryniewicz et al.^34^ in a solution containing 100 mmol L^–1^ pyrrole monomer (98%, Sigma-Aldrich), 5 mmol L^–1^ methyl orange (Synth) and 8 mmol L^–1^ KNO_3_ (Sigma-Aldrich) as electrolytic medium. The pH of the solution was adjusted to 2 with 1 mol L^–1^ HNO_3_ (Synth) 2 at 25 °C. The electropolymerization was performed by applying a constant potential of 0.8 V and a load control of 1000 mC cm^–2^, aiming to ensure the same amount of polypyrrole nanotubes (PPy-NTs) in the different electrodes.

Commercially available carboxylated multiwalled nanotubes were dispersed in isopropanol through sonication at 35% amplitude for 90 min at room temperature, with a concentration of 0.05% by weight. Subsequently, 2 μL of this MWCNTs solution was deposited onto the PPy-NTs modified electrode surface by drop casting and dried overnight.

Initially, carboxyl groups were activated using EDC (Sigma-Aldrich)/NHS (Sigma-Aldrich) (10:15 mmol L^–1^) solution in PBS (Sigma-Aldrich) for 30 min to ensure covalent bonding with the bioreceptor. Subsequently, EGF antibodies were immobilized onto the working electrode surface by applying 15 μL of the 1000 ng mL^–1^ antibody solution and incubating it for 60 min. To prevent nonspecific interactions by deactivating any unbound reactive groups, 15 μL of a 1% BSA (Sigma-Aldrich) solution in PBS was added and incubated for 45 min. EIS measurements characterized all biosensor construction steps. Finally, the modified electrode was prepared for exposure to various concentrations of commercial EGF solutions and serum samples.

### Electrochemical and SEM Characterizations (mel)

The modified electrodes were characterized by several electrochemical and morphological techniques. Scanning Electron Microscopy (SEM) analysis was performed employing a TESCAN MIRA3 instrument to examine the surface morphology and structural changes induced by the functionalization process. Cyclic voltammetry (CV) measurements were conducted within a potential window of −0.5 to 0.5 V, using a scan rate of 20 mV s^–1^, to evaluate the electrochemical behavior and redox properties of modified surfaces. The EIS technique was also carried out by applying an amplitude of 10 mV around the open circuit potential (OCP), covering a frequency range from 10 kHz to 0.1 Hz in PBS solution.

### Biosensor Analytical Performance

For evaluation, the biosensors were previously characterized by EIS measurements with an amplitude of 10 mV around the OCP with a frequency range of 10 kHz to 0.1 Hz in PBS pH 7.4. To assess the detection effectiveness of EGF, the biosensors were exposed to different concentrations of Recombinant Human EGF protein (hEGF), produced by the Proteomics and Protein Engineering Laboratory of Instituto Carlos Chagas,^35^ diluted in PBS. Tested concentrations were 0.5, 5, 20, 40, 80, and 200 pg mL^–1^. Then, 20 μL of each solution was added to the electrode and incubated for 15 min.

Subsequently, the biosensor was thoroughly washed with PBS solution, and the EIS analysis was executed.

The calibration curve was constructed using the variation in the charge transfer resistance (Δ*r*_ct_). These values were calculated for each concentration and represent the difference between the *r*_ct_ obtained after incubation with the hEGF solutions and the *r*_ct_ of the biosensor in the BSA-blocked surface stage.

### Detection in Serum Sample

Serum samples were obtained from a previous study in collaboration with the Brazilian National Cancer Institute (INCA) from metastatic lung adenocarcinoma patients. All patients signed informed consent forms, and the use of clinical samples was approved by the Ethics Committee (CAAE: 31531314.4.1001.5274). The samples were stored at −80 °C until analyzed. The samples were centrifuged at 4 °C, at 20.000 rpm for 15 min before use, to remove particles from the solution that could influence the analysis.

Serum samples were obtained from 43 metastatic lung adenocarcinoma patients; 15 of these patients’ serum samples were also obtained after the chemotherapy treatment started (Tables S1–S2).

The concentration of human EGF in serum was quantified using a Human EGF Quantikine enzyme-linked immunosorbent assay (ELISA) Kit (DEG00, R&D Systems) according to the manufacturer’s instructions. Briefly, the serum samples (diluted with the kit diluent at 1:10 as the starting point) were added to 96-well microplates previously coated with monoclonal antibody against human EGF and incubated at room temperature for 2 h. After the plate washing, the horseradish peroxidase–conjugated EGF antibody was added to the wells, and after 2h of incubation and washing, the substrate solution was added, and the plate was incubated for an additional 20 min protected from light. The reaction was stopped by the addition of the stop solution. After incubation, the optical density was measured at 450 nm with the correction wavelength set at 570 nm using a multimode microplate reader Synergy H1 (BioTek). The concentration was calculated from a standard curve using 4PL at GraphPad Prisma. The detection limit was 3.9 pg mL^–1^.

To test the ability of the electrochemical biosensor to detect positive clinical samples, 36 of the 58 serum samples were diluted in PBS at a 1:100 dilution factor, and 15 μL of this solution was deposited on the biosensor, after a blank EIS measurement, which is the measurement of the biosensor prepared before the addition of the analyte. EIS was performed with an amplitude of 10 mV around the OCP with a frequency range of 10 kHz to 0.1 Hz in PBS pH 7.4. The sample incubation time was 15 min. After washing with PBS for 5 min, EIS measurements were reperformed.

### Statistical Analysis

Regression analysis was conducted to compare data obtained from enzyme-linked immunosorbent assay (ELISA) and electrochemical biosensor measurements. The degree of agreement between the electrochemical biosensor and the reference method was assessed using Pearson correlation and Bland-Altman analysis. The differences between the two methods were plotted against their mean values. The limits of agreement, defined as the mean difference ± 1.96 standard deviations (SD) of the differences, were calculated to evaluate whether the agreement between the methods was acceptable.^36^ These limits are expected to encompass 95% of the measured values, indicating good agreement.^37,38^

The ELISA measurements were analyzed using BioRender Graph and or GraphPad Prism5. The differences were calculated by an unpaired *t* test for all the results or a paired *t* test when comparing the same patient measurement before and during the treatment.

## Results and Discussions

### Biosensor Construction and Characterization

The electrodes were analyzed using SEM to investigate their morphological characteristics, as presented in Figure 1a,b. In Figure 1a, the characteristic nanotubular morphology of PPy-NTs is observed, indicating the deposition of these structures on the electrode surface. Furthermore, Figure 1b illustrates the well-distributed arrangement of MWCNT-COOH on the PPy-NTs surface, forming a uniform mesh-like structure.

**Figure 1:**
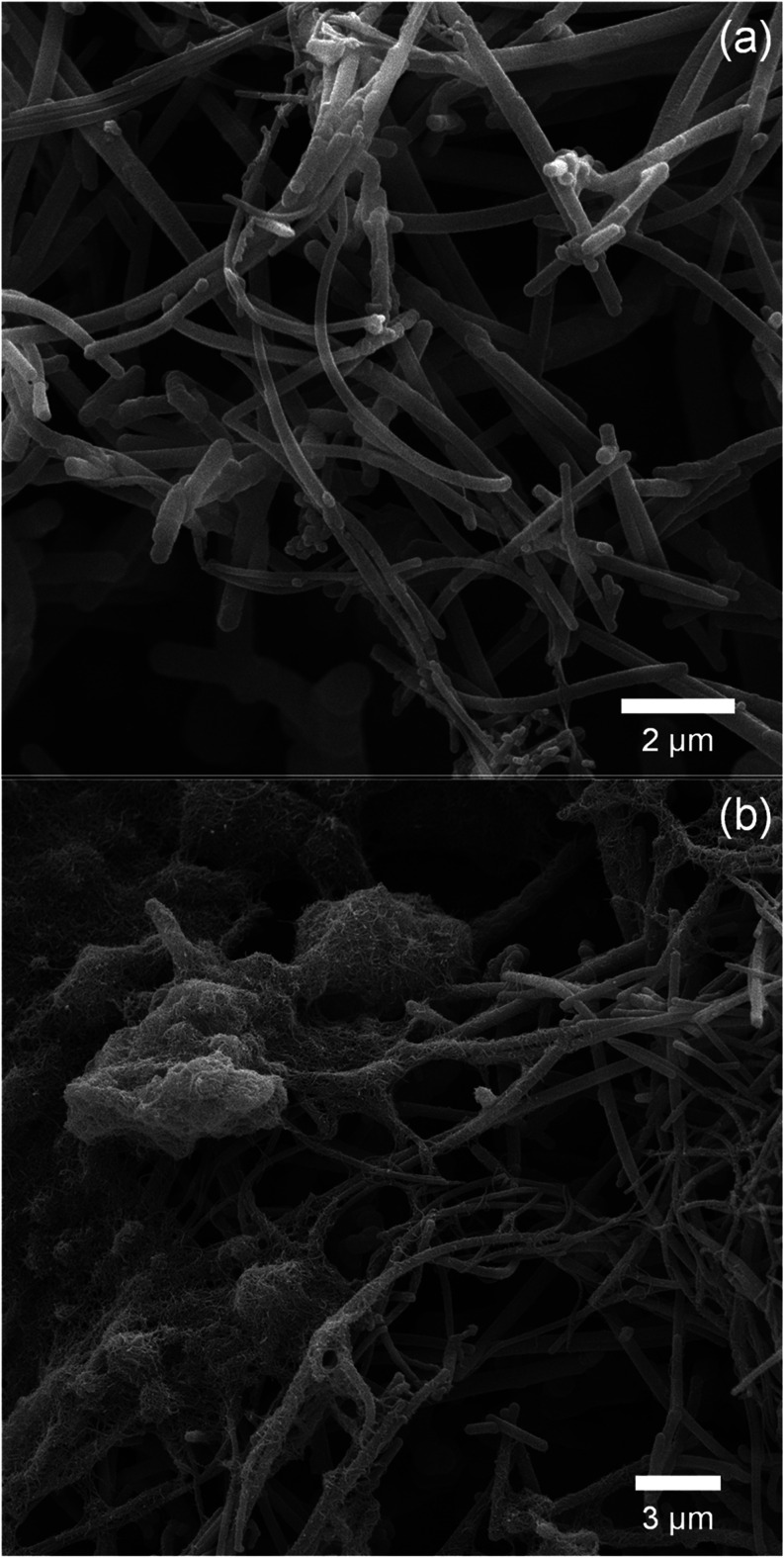
Representative SEM images of (a) PPy-NTs and (b) PPy-NTs/MWCNT.

The electrochemical properties of the electrodes were evaluated through CV, as shown in Figure 2a. The CV profile presented broad redox peaks and a large capacitive current, which are typical features of PPy-NTs.^34^ Additionally, the incorporation of MWCNT-COOH led to a slight reduction in current intensity.

**Figure 2:**
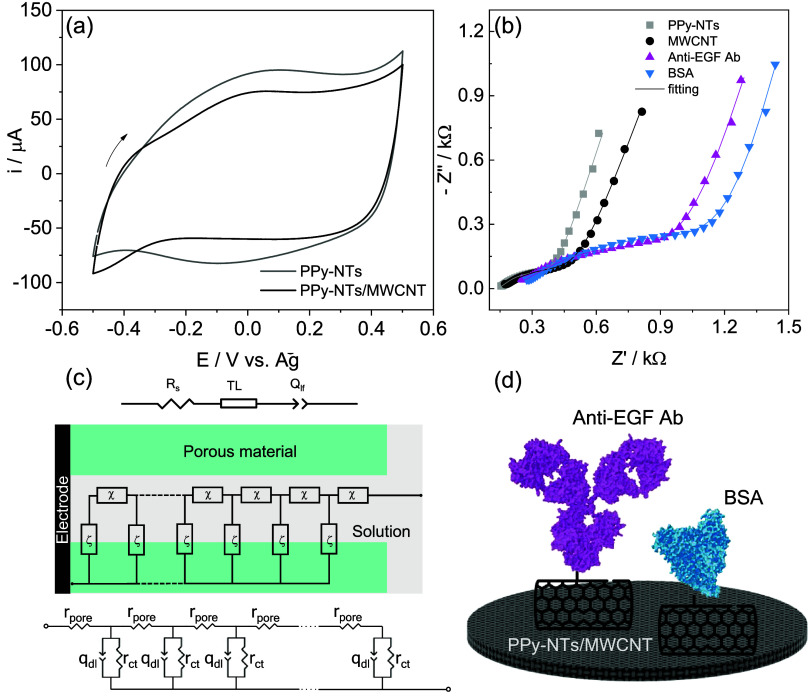
(a) Cyclic voltammetry at 20 mV s^–1^, (b) Nyquist diagram of PPy-NTs and PPy-NTs/MWCNT. Electrolyte = PBS pH 7.4. (c) Mixed TL and equivalent circuit model used to fit the EIS data from Figure (b). TL encompasses a single-channel model with distributed elements of impedance representing solution inside the pore (χ) and the electrode/electrolyte interface (ζ). (d) Scheme of the electrode surface modified with PPy-NTs/MWCNT, anti-EGF Ab and BSA.

The modified electrodes were also characterized by EIS, as depicted in the Nyquist plot in Figure 2b. Due to the porous structure of the PPy-NTs and MWCNT hybrid material, the EIS data were fitted using a mixed model that combined an equivalent circuit and a single-channel transmission line (TL) (Figure 2c).^39^ This model accounts for the influence of the electrolyte within the pores on the impedance response, providing a more accurate interpretation of the data.^40–42^ The equivalent circuit includes a series resistance (*R*_s_), representing the resistance of the electrolyte, cables, and current collector, along with a constant phase element (CPE) (*Q*_lf_), which is associated with charge accumulation in the polymeric film to maintain charge neutrality during redox reactions. These components are connected in series with the TL model, which comprises two distributed elements: χ (Ω m^–1^), representing the potential drop inside the pores and modeled by a pore resistance (*r*_pore_), and ζ (Ω m), related to processes at the electrode/electrolyte interface, represented by a charge transfer resistance (*r*_ct_) in parallel with another CPE (*q*_dl_), which reflects the double-layer capacitance due to charge accumulation at the interface (Figure 2c). The fitting parameters are listed in Table 1. The incorporation of MWCNT resulted in a significant increase in *r*_ct_ and *r*_pore_ values and a decrease in the *Q*_lf_ value. These results suggest that MWCNT partially obstructs the electroactive sites of PPy-NTs, hindering the charge transfer process and charge counterbalance, while also compromising ionic conductivity within the pores, which is in agreement with previous findings.^1,30^ The remaining parameters were not significantly altered by the addition of MWCNT.

**Table 1:** Parameters Obtained by Fitting EIS Data from Figure 2b Using the Mixed TL/Equivalent Circuit Model From Figure 2c

sample	*R*_s_/Ω	*r*_pore_/Ω	*r*_ct_/Ω	*Q*_dl_/mF s^n–1^	*n*_dl_	*Q*_lf_/mF s^n–1^	*n*_lf_
PPy-NTs	132	70	263	0.29	0.54	1.94	0.81
MWCNT	127	128	380	0.31	0.46	1.70	0.78
anti-EGF Ab	163	266	856	0.16	0.48	1.53	0.83
BSA	178	303	1043	0.15	0.48	1.49	0.86

The biosensor was constructed initially by activating the carboxyl groups from MWCNT using EDC/NHS coupling reaction, followed by immobilization of anti-EGF Ab for specific immunorecognition and BSA as a blocking agent (Figure 2d). Each step of biosensor construction was characterized by EIS, as shown in Figure 2b. After the immobilization of anti-EGF Ab and BSA (Figure 2d), an increase in both *r*_pore_ and *r*_ct_ values is observed, as these proteins can obstruct the material pores and block some electroactive sites at the electrode/electrolyte interface. This effect may also contribute to the reduction in *Q*_lf_ values, since charge transport in and out of the polymeric matrix becomes more restricted. Furthermore, *q*_dl_ values decrease after each modification step, indicating that the electroactive surface area progressively diminishes as biomolecules are immobilized on the electrode surface.

### Biosensor Analytical Evaluation

The biosensor performance was evaluated by EIS in PBS solution after sequential 15 min incubations at increasing concentrations of the EGF biomarker. After each incubation step, the electrode was briefly washed with PBS to remove unbound species. The impedance measurement required approximately 5 min, allowing the sensor response to be obtained within a total analysis time of about 20 min. Nyquist plots show an increase in *r*_ct_ after each incubation step, as illustrated in Figure 3a. The EIS data were fitted using the equivalent circuit shown in Figure 2c, and Δ*r*_ct_ was calculated for each concentration. The mean variation of Δ*r*_ct_ with EGF concentration is presented in Figure 3b, along with standard deviations (*n* = 5). A wide linear range from 500 fg mL^–1^ to 200 pg mL^–1^ with high linearity (*R* ^2^ = 0.9924) was obtained with the linear equation Δ*r*_ct_ = 36.49 + 81.35 logC_EGF_, and a limit of quantification (LOQ) was determined experimentally as the first concentration in the linear range (500 fg mL^–1^). LOD was calculated as the concentration producing a Δ*r*_ct_ equal to 3 times the noise (3:1 signal-to-noise ratio) using the standard deviation of blank samples (*n* = 6), and a value of 392 fg mL^–1^ was obtained. The reproducibility of the developed biosensors was evaluated from the calibration curve experiments. Each concentration was measured in five different electrodes, and the relative standard deviation (RSD) values ranged from 0.09 to 8.40%, demonstrating good reproducibility of the biosensing platform. The storage stability of the developed biosensor was evaluated over 10 days. All biosensors were fabricated on the same day and stored at 4 °C in PBS. Over the days, individual sensors were used to measure the electrochemical response to EGF, and the results were compared with the response of a freshly prepared electrode. The results showed that after 10 days, the biosensor retained approximately 75% of its initial response, indicating an operational lifetime of about 8 days for the sensing platform.

**Figure 3:**
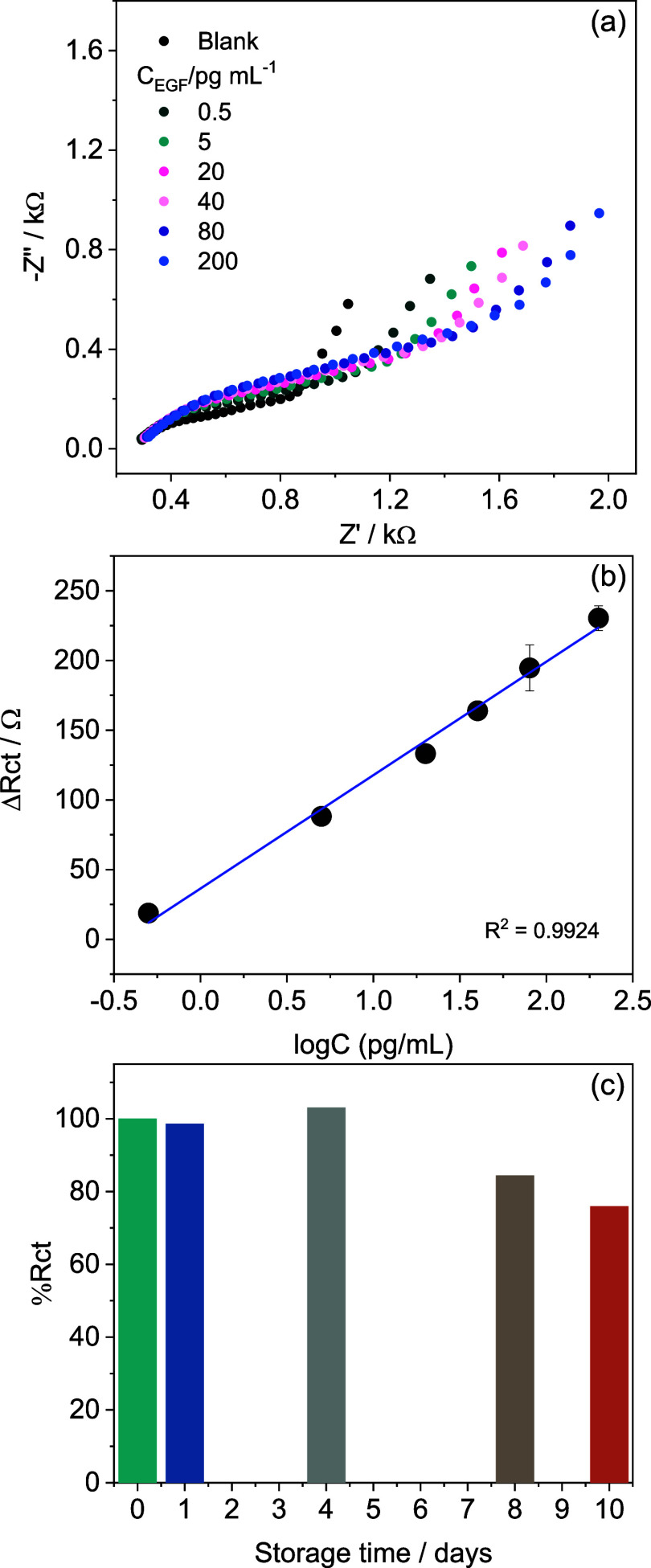
(a) EIS response of the biosensor after incubation with different concentrations of EGF in PBS and (b) the corresponding analytical curves. Error bars represent standard deviations obtained for five measurements on different electrodes (*n* = 5). (c) Storage stability of the developed EGF biosensor over 10 days. Biosensors were fabricated on the same day and stored at 4 °C in PBS. The electrochemical response to EGF was compared with that of a freshly prepared electrode.

The remarkable low detection limit can be attributed to the use of EIS as a label-free detection technique, which is inherently more sensitive to changes at the electrode surface compared to commonly employed techniques such as voltammetric ones at the electrode surface compared to commonly employed techniques such as voltammetric ones. Furthermore, the use of a nanostructured material increases the surface area available for antibody immobilization, further contributing to improved performance.

At the time of publication, this research represents the first impedimetric biosensor developed for the direct and highly sensitive detection of EGF protein in the serum of patients with lung adenocarcinoma. Research found in the literature has focused on the electrochemical detection of epidermal growth factor receptor (EGFR) mutations and related DNA sequences in biological samples. It is important to highlight that EGFR ligands, such as EGF, can be secreted into the tumor microenvironment and circulate in biological fluids, enabling their detection in serum samples. Consequently, monitoring circulating EGF levels may provide valuable information about the activation state of the EGFR signaling pathway and disease progression, complementing approaches focused exclusively on receptor mutations or overexpression. In contrast, many EGFR-based detection strategies rely on analyzing tumor tissue or cellular material, frequently obtained through biopsy procedures, or on detecting EGFR mutations in ctDNA in blood; however, EGFR mutations occur in approximately 10–40% of cases of EGFR deregulation, whereas EGFR overexpression has been reported in up to 85% of cases.^43–47^ An example of such work is that of Tian et al.,^48^ who developed a paper-based electrochemical DNA biosensor for EGFR mutations, achieving a detection limit of 0.167 nM. Similarly, Elshafey et al.^49^ developed an impedimetric immunosensor for the cancer marker EGFR utilizing gold nanoparticles and protein G, reporting a detection limit of 0.34 pg mL^–1^ in PBS and 0.88 pg mL^–1^ in human plasma. Vasudev et al.^46,50^ developed a label-free electrochemical immunosensor for the detection of epidermal growth factor receptor (EGFR) using antibodies immobilized in a self-assembled monolayer on gold electrodes, achieving a detection limit of 1 pg mL^–1^. Ganbold et al.^51^ developed an aptamer-based capacitive biosensor for EGFR detection using a Cr/Au interdigitated electrode modified with a self-assembled monolayer of 3-mercaptopropionic acid (MPA) for aptamer immobilization. This platform achieved a detection limit of 0.005 ng mL^–1^ for EGFR, demonstrating its potential for the early detection of nonsmall cell lung cancer. More recently, advances in electrochemical biosensor platforms have demonstrated improved analytical performance and applicability for the detection of clinical biomarkers in complex biological matrices. Compared with these approaches, the biosensor developed in this work directly detects EGF protein, a ligand linked to lung cancer progression and response to treatment. The LOD of 392 fg mL^–1^ achieved is very promising and, in most cases, exceeds the LOD reported for electrochemical biosensors detecting EGFR, especially considering the difference in the nature and size of the target analyte (ligand protein vs DNA/receptor), in addition to successful validation using serum samples from patients with lung adenocarcinoma. This approach offers a novel diagnostic and prognostic tool, particularly relevant for assessing EGF levels in the context of emerging therapies, such as the CIMAvax-EGF vaccine.

### Detection of EGF in Serum Samples

EGF levels in the serum of 43 individuals diagnosed with NSCLC were initially evaluated by ELISA. A wide range of EGF concentration was observed in samples from patients before starting the chemotherapy treatment (7.6–740.4 pg mL^–1^) with a mean of 275.7 pg mL^–1^ ± 30.6 (SEM SDEM, Standard error of the mean error) (Tables S1–S2 and Figure 4a). Serum samples were also obtained from a group of 15 patients who started therapy. In these samples, EGF levels varied from 15.7 to 522.7 pg mL^–1^ (mean 164.4 SDEM 35.3). When comparing the data from the same patients, before and after receiving treatment, it is possible to observe a decreasing trend in EGF levels, although the difference does not reach statistical significance. Notably, in samples with higher levels of EGF before the therapy (above 300 pg mL^–1^), a significant reduction in EGF levels was observed (Figure 4b). EGF concentration in human serum has been shown to be largely variable, both in healthy and lung cancer patients, and factors such as age,^52^ tumor stage or presence of metastasis^8,10,11^ can influence its concentration, potentially contributing to discrepancies reported across different studies. Despite the limited number of samples, our data indicates that for patients with high EGF levels, the start of a standard treatment for adenocarcinoma might lead to a reduction in EGF levels (Figure 4d), while less variation occurs in patients with lower pretreatment EGF levels (Figure 4c).

**Figure 4:**
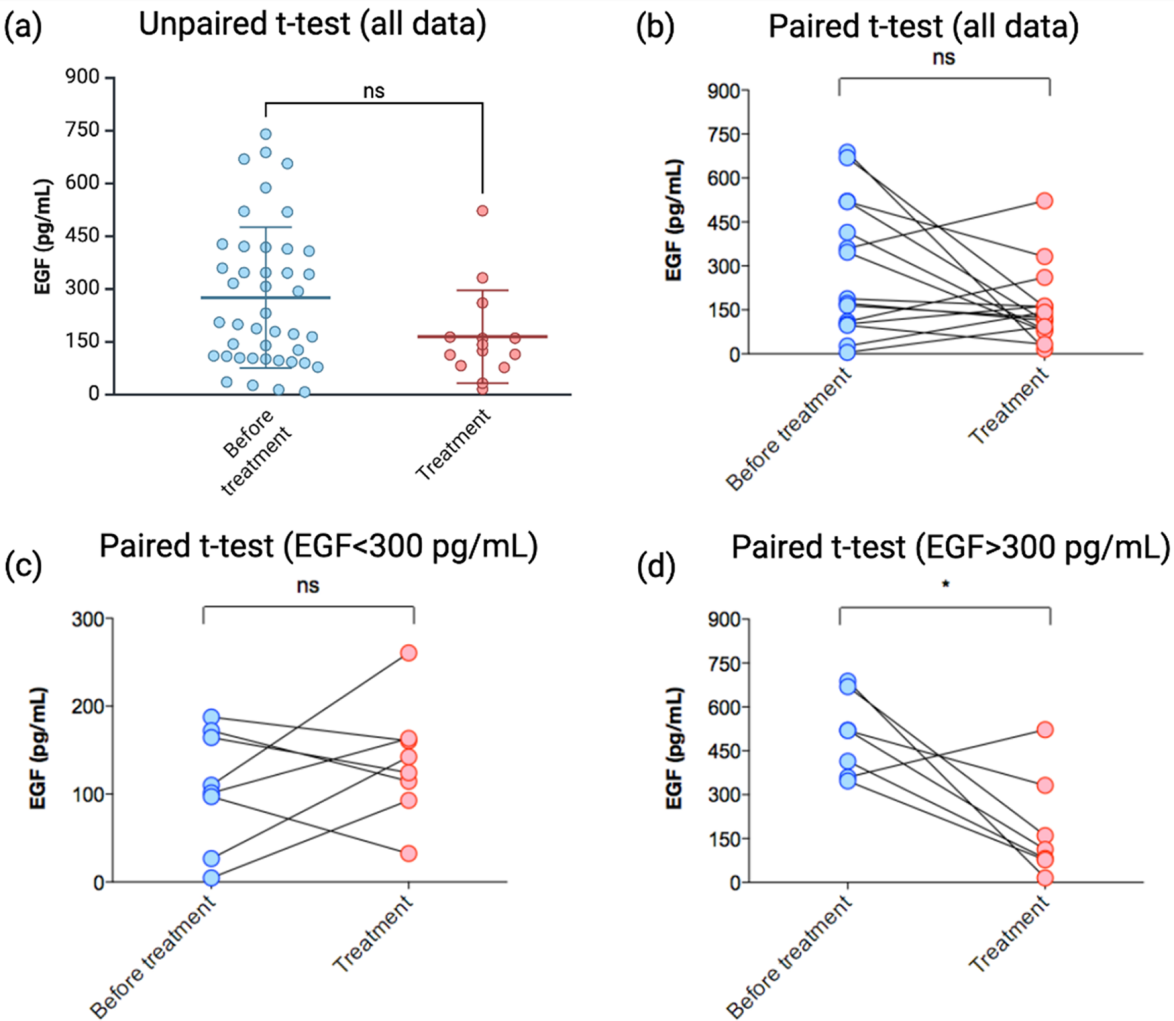
Quantification of EGF by ELISA. (a) The concentration range of EGF varied from 7.6–740.4 pg mL^–1^ in the serum of adenocarcinoma patients before any therapy intervention. For those who started treatment, the EGF levels ranged from 15.7 to 522.7 pg mL^–1^. (b) Although treatment indicates a tendency to decrease, the difference is not significant. When analyzed separately, less variation was observed for patients with lower pretreatment EGF (c), while the group of patients with high pretreatment EGF (above 300 pg mL^–1^) showed a significant reduction after treatment (d).

To evaluate the efficacy of our biosensor in detecting EGF levels in clinical samples, the samples evaluated by ELISA were analyzed by the electrochemical biosensor. EGF levels found by the electrochemical biosensors were compared with the concentrations found using ELISA. To determine if there is a matrix effect from diluted serum samples (1:100 dilution) in the biosensor analyses, different concentrations of EGF were spiked into a serum sample with EGF level determined by ELISA assay much lower than our LOQ at a 1:100 dilution (0.046 pg mL^–1^), which is considered negative by our biosensor. The recoveries obtained for two different target concentrations in spiked samples were 120% (500 fg mL^–1^) and 93.7% (20 pg mL^–1^). These results confirm that the assay demonstrates high accuracy in detecting EGF in serum samples, with no significant matrix effects or interference observed.

In this way, serum samples from lung adenocarcinoma patients were tested using the electrochemical biosensor at a 1:100 dilution. The EGF concentrations measured by our biosensor were compared to those obtained from ELISA, as demonstrated in Figure 5a. Pearson’s correlation analysis showed a strong association between the EGF concentrations obtained from the ELISA kit and those from the electrochemical biosensor (*r* = 0.865, *p* < 0.0001), further confirming the high accuracy of the developed sensing platform. Furthermore, based on the concentration values obtained from ELISA for all samples, EGF levels were undetectable in only a small percentage (12%) at a 1:100 dilution using our biosensor. Most of these samples returned negative results with our biosensor and were not displayed in Figure 5a. It is noteworthy that elevated EGF levels are linked to NSCLC and other diseases, while low EGF levels are associated with a more favorable prognosis.^3^ Although our study does not aim to establish direct comparisons with the broader population and is based on a limited sample size, our findings indicate a trend toward lower EGF concentrations for patients who underwent chemotherapy.

**Figure 5:**
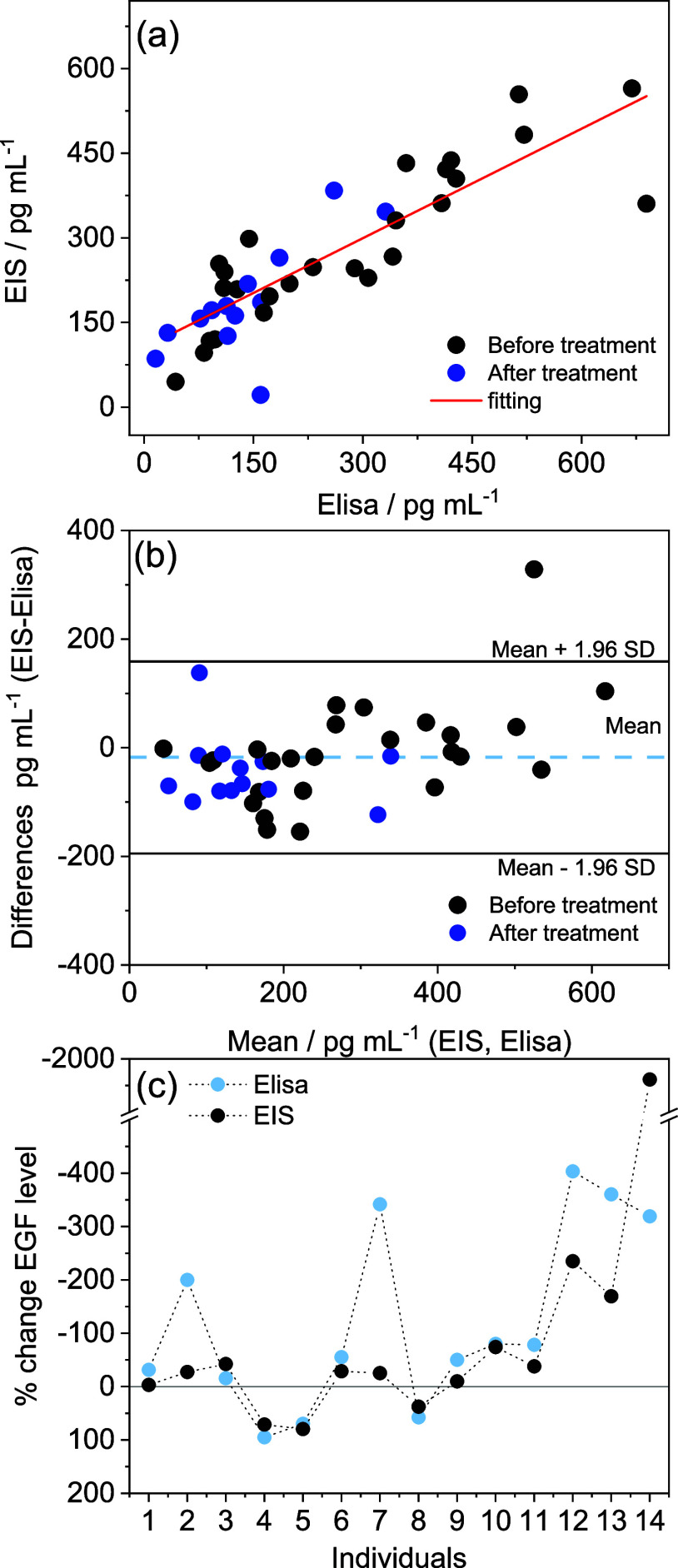
(a) Pearson’s correlation analysis for the association between EGF concentrations measured by the ELISA kit and the electrochemical biosensor. (b) Bland–Altman plot assessing the level of agreement between the electrochemical biosensor and the ELISA method. (c) Percentage change in EGF levels following treatment, relative to pretreatment levels, determined by both ELISA and the electrochemical biosensor.

Bland-Altman analysis quantifies the agreement levels between the electrochemical biosensor and the reference method by emphasizing any biases and differences between the two measurement methods. Bland–Altman plot revealed that 97.4% of the differences between the measured values obtained from the ELISA test kit and the developed biosensor fell within the limits of agreement (Figure 5b), demonstrating no statistically significant difference between the two methods.

The percentage change in EGF levels following treatment was calculated relative to pretreatment levels, using EGF concentrations determined by both ELISA and the electrochemical biosensor. A comparison between both methods is presented in Figure 5c. A similar change in EGF levels was observed for each sample using both methods, indicating that the electrochemical biosensor could be a feasible alternative for evaluating EGF levels in clinical samples.

## Conclusion

The work developed a new impedimetric biosensor based on PPy-NTs and MWCNT-COOH modified with anti-EGF antibodies for the sensitive and accurate detection of EGF in serum samples from patients with lung adenocarcinoma. The biosensor demonstrated a LOD of 392 fg mL^–1^ and a wide linear range, evidencing its high sensitivity. Bland-Altman analysis and Pearson’s correlation of the biosensor and the ELISA method confirmed a strong agreement and the absence of statistically significant differences between them. Furthermore, data obtained from both ELISA and the electrochemical biosensor suggest a trend toward reduced EGF concentrations in patients who underwent chemotherapy, although the study is based on a limited sample size and, therefore, may not be representative of a broader population. The biosensor presents itself as a promising and reliable alternative for monitoring EGF levels, with the potential to aid in the evaluation of the efficacy of antineoplastic treatments and in the prognosis of the disease, being one of the first studies to explore the detection of EGF in patients with lung cancer using an impedimetric biosensor platform.

## Supplementary Material



## Notes

Serum EGF levels, determined by ELISA, in 43 individuals diagnosed with NSCLC (Table S1); serum EGF levels, determined by ELISA, in individuals diagnosed with NSCLC, with serum samples collected before and after chemotherapy (Table S2) (PDF)
