# Supplementary material for: Sensitive Detection of Epidermal Growth Factor in Lung Cancer Patients by Electrochemical Biosensors
Source: Anal Chem. 2026 Apr 24;98(18):13940–9. doi: 10.1021/acs.analchem.6c01773 (PMC13177281; doi:10.1021/acs.analchem.6c01773)
Supplement: Supplementary file 1 [file ac6c01773_si_001.pdf]

Sensitive detection of epidermal growth factor in lung cancer patients by electrochemical biosensors

Tatiana Lima Valério[a], Bruna M. Hryniewicz[b*], Fernanda Luisa Basei[c], Mel De Souza Wendhausen Araújo[a], Hussamaldeen Jaradat[d], Olfa Kanoun[d], Flavia Raquel Gonçalves Carneiro[efg*], Tatiane Caldas Montella[h], Carlos Gil Moreira Ferreira[h], Nilson Ivo Tonin Zanchin[c], Marcio Vidotti[a*]

*mvidotti@ufpr.br

*brunahryniewicz@ufba.br

[a] Grupo de Pesquisa em Macromoléculas e Interfaces, Federal University of Paraná (UFPR), 81531-980, Curitiba, PR, Brazil

[b] Departamento de Físico-Química, Instituto de Química, Universidade Federal da Bahia, Salvador, Bahia 40170-115, Brazil

[c] Instituto Carlos Chagas – Fiocruz, 81310-020, Curitiba, PR, Brazil

[d] Professorship of Measurement and Sensor Technology, Faculty of Electrical Engineering and Information Technology, Chemnitz University of Technology, 09126, Chemnitz, Germany

[e] Center for Technology Development in Health (CDTS), FIOCRUZ, Rio de Janeiro 21040-900, RJ, Brazil

[f] Interdisciplinary Laboratory of Medical Research, Oswaldo Cruz Institute (IOC), FIOCRUZ, Rio de Janeiro 21040-900, RJ, Brazil

[g] Program of Immunology and Tumor Biology, Brazilian National Cancer Institute (INCA), Rio de Janeiro 20231-050, RJ, Brazil

[h] Oncoclínicas, Rio de Janeiro 22250-905, Brazil

Supporting Information

Table S1. Serum EGF levels, determined by ELISA, in 43 individuals diagnosed with NSCLC.

ID	EGF level (pg/mL)	SD	Treatment
001	740,4	27,9	n
002	688,4	28,9	n
003	669,7	5,5	n
004	656,7	21,5	n
005	588,0	3,7	n
006	521,1	4,7	n
007	519,3	7,4	n
008	427,9	7,1	n
009	421,2	3,8	n
010	418,8	58,4	n
011	414,3	10,5	n
012	408,0	24,4	n
013	359,5	32,9	n
014	346,9	5,6	n
015	346,8	40,2	n
016	345,7	14,5	n
017	342,1	10,5	n
018	316,4	2,1	n
019	307,5	24,5	n
020	293,6	4,5	n
021	231,6	6,2	n
022	205,5	4,4	n
023	199,5	13,3	n
024	187,7	2,2	n
025	179,1	9,3	n
026	172,2	1,3	n
027	164,3	6,3	n
028	143,9	2,7	n
029	139,7	1,1	n
030	126,8	4,8	n
031	110,2	1,8	n
032	109,2	0,6	n
033	104,5	1,7	n
034	103,3	2,3	n
035	101,5	7,7	n
036	97,2	3,5	n
037	89,9	4,1	n
038	78,2	1,8	n
039	35,8	1,4	n
040	26,5	1,8	n
041	14,2	0,6	n

042	7,6	0,3	n
043	4,6	0,2	n
035	522,7	7,7	y
013	331,9	4,4	y
011	260,6	5,6	y
006	163,4	5,8	y
003	160,5	0,6	y
002	159,8	2,2	y
027	142,3	3,3	y
036	124,4	4,1	y
024	114,7	3,2	y
043	113,2	6,9	y
040	92,9	1,3	y
007	82,3	5,4	y
015	77,2	0,6	y
031	32,4	1,1	y
026	15,7	0,1	y

Table S2. Serum EGF levels, determined by ELISA, in individuals diagnosed with NSCLC, with serum samples collected before and after chemotherapy.

	**Before treatment**		**Treatment**	
**ID**	**EGF level (pg/mL)**	**SD**	**EGF level (pg/mL)**	**SD**
002	688,4	28,9	15,7	0,1
003	669,7	5,5	159,8	2,2
006	521,1	4,7	113,2	6,9
007	519,3	7,4	331,9	4,4
011	414,3	10,5	82,3	5,4
013	359,5	32,9	522,7	7,7
015	346,8	40,2	77,2	0,6
024	187,7	2,2	160,5	0,6
026	172,2	1,3	114,7	3,2
027	164,3	6,3	124,4	4,1
031	110,2	1,8	260,6	5,6
035	101,5	7,7	163,4	5,8
036	97,2	3,5	32,4	1,1
041	26,5	1,8	142,3	3,3
043	4,6	0,2	92,9	1,3
